# Demography and space‐use of Eastern Red‐backed Salamanders (*Plethodon cinereus*) between mature and successional forests

**DOI:** 10.1002/ece3.9764

**Published:** 2023-01-24

**Authors:** Meaghan R. Gade, Philip R. Gould, Andrew J. Wilk, Kate C. Donlon, MacKenzie L. Brown, Marnie L. Behan, Marissa A. Roseman, Annalee M. Tutterow, Evan D. Amber, Ryan B. Wagner, Andrew S. Hoffman, Jennifer M. Myers, William E. Peterman

**Affiliations:** ^1^ School of Environment and Natural Resources The Ohio State University Columbus Ohio USA; ^2^ Department of Ecology and Evolution Yale University New Haven Connecticut USA

**Keywords:** demography, fecundity, fine‐scale, forest, salamander, succession

## Abstract

Space‐use and demographic processes are critical to the persistence of populations across space and time. Despite their importance, estimates of these processes are often derived from a limited number of populations spanning broad habitat or environmental gradients. With increasing appreciation of the role fine‐scale environmental variation in microgeographic adaptation, there is a need and value to assessing within‐site variation in space‐use and demographic patterns. In this study, we analyze 3 years of spatial capture–recapture data on the Eastern Red‐backed Salamander collected from a mixed‐use deciduous forest site in central Ohio, USA. Study plots were situated in both a mature forest stand and successional forest stand separated by <100‐m distance. Our results showed that salamander density was reduced on successional plots, which corresponded with greater distance between nearest neighbors, less overlap in core use areas, greater space‐use, and greater shifts in activity centers when compared to salamanders occupying the mature habitat. By contrast, individual growth rates of salamanders occupying the successional forest were significantly greater than salamanders in the mature forest. These estimates result in successional plot salamanders reaching maturity more than 1 year earlier than salamanders on the mature forest plots and increasing their estimated lifetime fecundity by as much as 43%. The patterns we observed in space‐use and individual growth are likely the result of density‐dependent processes, potentially reflecting differences in resource availability or quality. Our study highlights how fine‐scale, within‐site variation can shape population demographics. As research into the demographic and population consequences of climate change and habitat loss and alteration continue, future research should take care to acknowledge the role that fine‐scale variation may play, especially for abiotically sensitive organisms with limited vagility.

## Graphical abstract

Time to maturity is a critical life history trait. In this study, we find that salamanders occupying mature and successional forest habitats separated by <100 m differed in their time to maturity by over a year. The delayed time to maturity of individuals in the mature forest results in a 43% reduction in lifetime fecundity for salamanders inhabiting the mature forest.

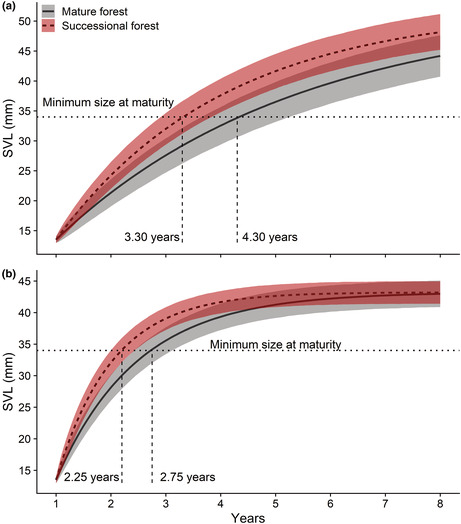

## 1 INTRODUCTION

Landscapes are composed of biotic and abiotic features that are heterogeneous in at least one dimension at any scale (Turner & Gardner, 2015). Extant populations of organisms are usually acclimated to their local landscapes (Urban et al., 2016); however, much of our knowledge about a given species is often derived from a limited number of populations and assume demographic processes are spatially invariant. Such generalizations may be particularly misleading for organisms that interact with their environment at fine scales (i.e., tens of meters or less), such as plants, invertebrates, and many amphibians because these organisms typically have limited dispersal and may have physiological and/or behavioral constraints that restrict their ability to actively select habitat (De Bie et al., 2012). It is therefore imperative to evaluate variation in demographic processes at spatial scales relevant to the target organisms to mitigate potential biases of broad‐scale generalizations.

An increasing body of evidence indicates that microgeographic adaptation occurs among numerous species and ecosystems (Richardson et al., 2014), influencing distribution, abundance, and individual phenotype (Cicchino et al., 2021). For example, limpets (genus *Patella*) separated by <2 m experienced significantly different sun exposure and thermal stress depending on the side of the rock that they inhabit (Seabra et al., 2011). Chronic thermal stress can significantly impact growth, reproduction, and overall fitness (Dantzer et al., 2014; Wingfield & Romero, 2011), which likely results in heterogeneous fitness among individuals in close proximity to each other. Similarly, high‐elevation hatchlings of the water snake, *Natrix maura*, are smaller in size and poorer swimmers compared to those at low elevations, likely as a result of hypoxia and lower temperatures (Souchet et al., 2020). Therefore, the evaluation of fine‐scale demographic differences between populations is a key step to understanding spatial nuance that may be important for the development of comprehensive conservation and management goals of organisms with limited habitat selection capability.

Space‐use, or the amount and extent of a given area used, by individuals within a population shapes demographic patterns and can be influenced by the availability of resources, appropriate microclimate, and intraspecific density (Gaillard et al., 2010; Morales et al., 2010). Population density may be particularly influential because of the many cues it signals. For example, high‐density areas may indicate good quality habitats that can support more individuals and may offer cooperation with conspecifics (e.g., antipredator grouping behavior, resource sharing, and cooperative breeding) and consequently promote site philopatry and reduced movement and space‐use (Clobert et al., 2009; Le Galliard et al., 2005). Conversely, high‐density areas may have higher competition for resources, mates, and more aggressive individuals thus promoting space‐use and movement away from the high‐density site (Clobert et al., 2009). Similarly, population demographic parameters such as individual growth rates can vary in density‐dependent ways whereby higher densities result in lower individual growth rates, due to fewer resources available to each individual, and allocation of obtained resources toward other processes like aggressive interactions and competition (Getz, 1996). Animal movement and space‐use vary across species geographic range (Boyle et al., 2009), but also between populations in close proximity especially when microhabitats differ (Gonzales et al., 2020; Reeve et al., 2009). Yet, measuring life history and space‐use at fine scales can be challenging due to cost, labor intensity, time, and methodological constraints. Thus, we lack a comprehensive understanding of fine‐scale population dynamics.

Plethodontid salamanders are a particularly well‐suited group to evaluate fine‐scale variation in key demographic patterns due to their high abundances across a wide geographic range (Petranka, 1998), generally low dispersal rates, and relative ease of repeated captures. Terrestrial lungless salamanders in the genus *Plethodon* are the most abundant vertebrate animals in many North American forests, accounting for more vertebrate biomass than any other taxa in these ecosystems (Burton & Likens, 1975; Semlitsch et al., 2014). *Plethodon* are highly philopatric and rely on cool and moist microhabitat conditions to facilitate cutaneous respiration. The Eastern Red‐backed Salamander, *Plethodon cinereus*, is one of the most abundant and widely distributed species, and while we have some understanding of demographic patterns across their geographic range (Anthony & Pfingsten, 2013; Nagel, 1977; Sayler, 1966), the fine‐scale demographic differences between populations separated by small distances are largely unknown. *Plethodon cinereus* is found across eastern North American forests but has the highest density in forests with greater percent canopy cover, larger trees, and with high densities of well‐decayed coarse woody debris (McKenny et al., 2006; Otto et al., 2014; Wilk et al., 2020). Forests with these attributes provide suitable cool and moist microhabitats and higher prey abundance for salamanders. However, habitat heterogeneity can exist across fine scales with variable conditions and resources within a small area. As such, salamander demographic patterns likely differ between local populations (Farallo & Miles, 2016) and targeted research to unveil these differences is necessary.

Here, we use a multiseason spatial capture‐mark‐recapture study to evaluate fine‐scale demographic rates of *P. cinereus* inhabiting sites with different forest compositions but separated by just 100 m. We predicted that salamanders occupying the two different forest types will exhibit differences in density, space‐use, and individual growth rates. Specifically, we expected that early‐successional forest habitat would be suboptimal to mature forest due to reduced stand density promoting warmer and drier microclimate and reduced aboveground biomass that may reduce invertebrate prey (McKenny et al., 2006). As such, successional stands were predicted to support fewer individual salamanders with larger home ranges and lower individual growth rates compared with salamanders occupying the mature forest habitat.

## 2 METHODS

### 2.1 Field sampling

**FIGURE 1:**
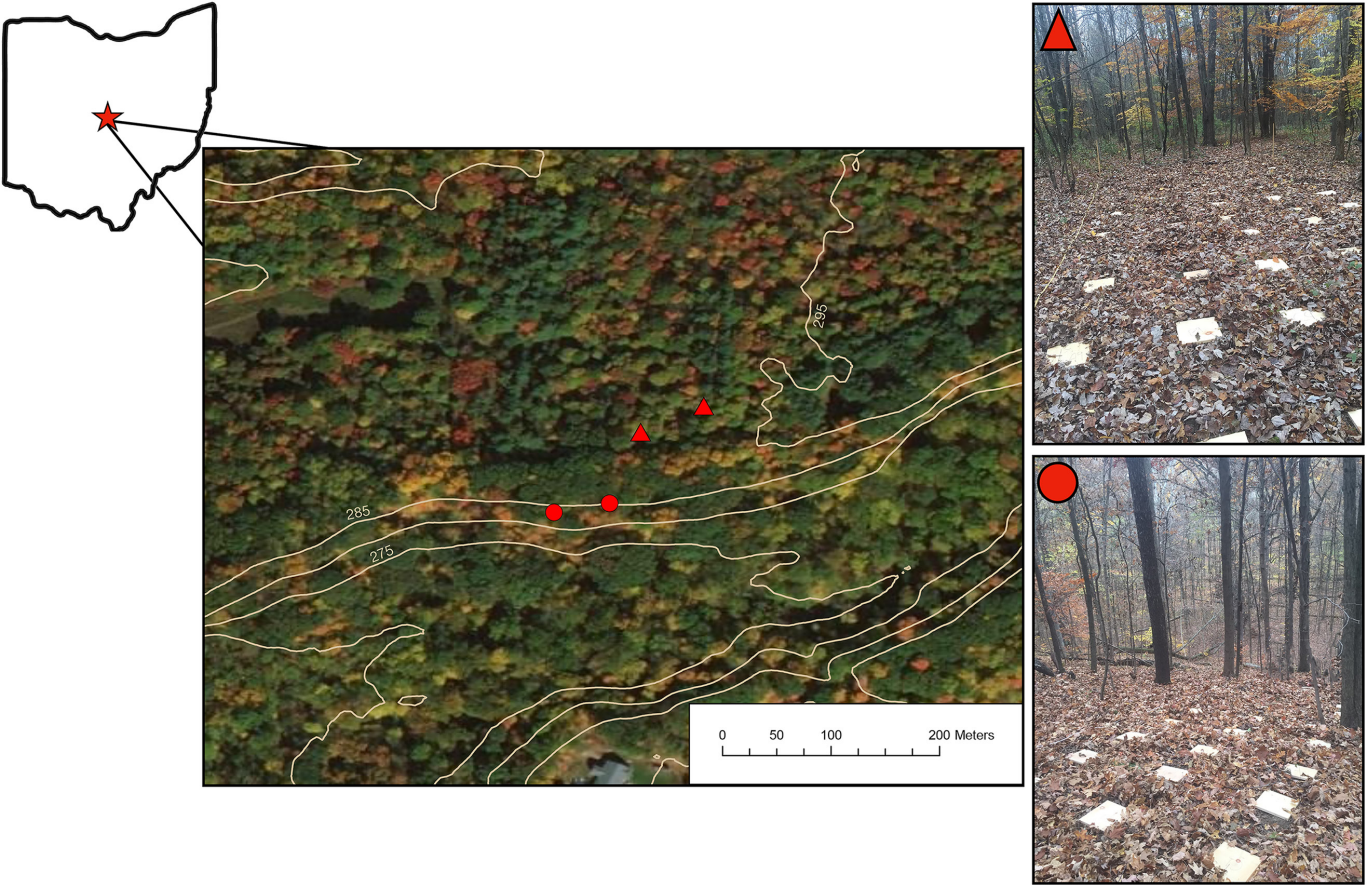
Map of the study site in Delaware County, Ohio, showing the location of the paired plots in successional (triangles) and mature (circles) forest. Inset images show associated coverboard arrays for each site.

We conducted this study in Galena, Ohio, at a 36‐ha site consisting of a mix of mature oak‐hickory forest (*Quercus* and *Carya* spp.), early‐ to mid‐successional mesic hardwoods (*Acer* spp.), white pine plantations (*Pinus strobus*), and open field habitats (Figure 1, Table 1). The early‐successional forests are <40 years in age and are growing in what was previously pastureland used for low‐intensity grazing in the 1970s. The mature forests are found on the ravine slopes and bottomlands surrounding a rocky stream that flows through the property. The well‐drained, relatively undisturbed upper slopes of this ravine are adjacent to the flat, poorly drained, early‐successional upland forests that were historically grazed.

**TABLE 1:** Mean ± standard deviation (SD) of the diameter at breast height (DBH, cm) and frequency of occurrence at successional and mature sites.

Tree	Mature		Successional	
	DBH	Count	DBH	Count
*Fagus* spp.	3.70 (1.07)	2	4.63 (0.68)	3
*Prunus* spp.	19.50 (18.10)	2	10.32 (5.71)	4
*Acer* spp.	7.69 (5.58)	46	18.15 (14.15)	32
*Quercus* spp.	35.36 (26.46)	16	16.20 (0)	1
*Carya* spp.	21.75 (7.09)	10		
*Pinus* spp.			56.10 (3.25)	2
*Platanus* spp.			20.20 (0)	1
*Fraximus* spp.			33.6 (8.91)	3
*Ulmus* spp			12.43 (5.75)	4
*Ostrya* spp.			4.05 (1.38)	7
	Mean = 17.60	Total = 76	Mean = 19.52	Total = 57

*Note*: All trees within a 10‐m radius of the center of each study plot were identified and measured.

We established four cover board arrays each consisting of wooden boards measuring 30 × 30 × 2.5 cm. We set two arrays in successional habitat and two arrays on the adjacent mature forest habitat. Each pair of arrays was at least 20 m apart and consisted of a 5 × 10‐m array of 50 cover boards equally spaced 1 m apart. Successional and mature forest arrays were 60–100 m apart. This design minimized distance between arrays while capturing perceived differences in abiotic gradients of temperature and moisture. We established all arrays during Fall 2016 and began sampling for salamanders in Spring 2017 after one season of board weathering. We identified all tree species and measured the diameter at breast height (DBH) in a 10‐m radius around the center of each array (Table 1).

*Plethodon cinereus* in Ohio are most active at the surface during spring and autumn and retreat into deeper soil to avoid desiccation and freezing during summer and winter, respectively (Anthony & Pfingsten, 2013). Thus, we sampled for salamanders three times during both the spring (March 15th–May 15th) and fall (September 15th–October 15th) from 2017 to 2019. During each daytime sampling event, we searched for salamanders under all cover boards, captured animals found underneath, and recorded the board and array of capture. We placed individual salamanders in zip‐top bags for processing. Each newly captured salamander was uniquely marked using a fluorescent subdermal visual elastomer implant, a technique that provides safe and long‐lasting marks (Grant, 2008; Northwest Marine Technologies, LLC). Each recaptured individual was identified by reading the visual elastomer implants with a UV flashlight. We also recorded snout–vent length (SVL, mm), tail length (TL), and sex of every individual. Males were determined based on the presence of a mental gland or visual testes, and females were determined by the presence of eggs (Gillette & Peterson, 2001). If neither were visible, we made an expert determination based on the shape of the nose as being either square (males) or round (females) (Dawley, 1992). Individuals were considered juveniles if they were <34 mm SVL (Anthony & Pfingsten, 2013) and were not assigned a sex. We returned salamanders to their board of capture within 4 h of initial capture.

Sampling occurred between 09:00 and 12:00 and during each sampling occasion, we measured weather covariates at each array including soil surface temperature, average leaf litter depth, and temperature. Additionally, we collected soil core samples at the center of each array to determine organic soil depth and obtain soil water content by measuring the difference in mass before and after air‐drying soil samples. We used t‐tests to determine statistical differences in these survey‐level measures between the successional and mature sites. We also retrieved weather covariates for each sampling survey for our study site from the PRISM dataset. For each year, we designated spring and fall as the active seasons, with the remainder of the year as the inactive seasons. We estimated average temperature, precipitation, and days since rain using a 5‐day moving window analysis for every day of active and nonactive seasons. We calculated the coefficient of variation for 5‐day average temperature and total precipitation by dividing the seasonal mean by the standard deviation.

### 2.2 Statistical analysis

#### 2.2.1 SCR model

We investigated survival and space‐use parameters using a robust‐design spatial capture–recapture (SCR) adapted from Ergon and Gardner (2014). The robust design describes a sampling structure that divides “primary” sessions and “secondary” sessions within each primary session. The robust design assumes that demographic processes are closed between secondary sessions but open between primary sessions (Pollock, 1982). In our study, fall and spring sampling seasons serve as the primary sessions with 2–3 secondary sessions within each primary session. An SCR differs from a traditional Cormack–Jolly–Seber capture–recapture model by explicitly incorporating spatial capture locations to account for individual movement or dispersal, allowing for a more accurate estimate of true survival (Schaub & Royle, 2014). Dispersal distance is an estimate of the difference between activity centers between seasons and activity centers were assumed to have a uniform distribution and dispersal only occurred between primary sessions. We included the aforementioned PRISM weather covariates in the survival submodel of the SCR; however, null models were better supported and we subsequently only report results from those models.

#### 2.2.2 Individual growth model

We estimated individual growth using Fabens capture–recapture growth model (Fabens, 1965). The growth function for individual *i* at time *t* was defined as:

$$\mathrm{SVL}0_{i,t}=\mathrm{SVL}0_{i,t-1}+\left\{L\left[\mathrm{SEX}\right]-\mathrm{SVL}0_{i,t-1}\times \left[1-\exp\left(-K_{i,t}\times \frac{I}{365}\right)\right]\right\}$$

where asymptotic size *L* was allowed to differ by sex and was estimated from a normally distributed prior with a mean of 48 and precision of 0.01, a prior based on estimates from Muñoz, Miller, et al. (2016). SVL0_*i*,*t*_ *i*s the size at first capture and follows a uniform distribution with a minimum of 10 and maximum of 60. We removed any observations where SVL at the final time step was less than SVL at the first time step, which would be due to measurement errors. *K* represents the individual growth rate, and *I* is the annual scaling interval between captures. We estimated *K* as a function of categorical plot position (POS; Mature or Successional) and *SEX* based on observed change in SVL of recaptured individuals across sampling periods.

$$K_{i,t}=\beta_{0}^{\left[K\right]}+\beta_{1}^{\left[K\right]}\times {\mathrm{POS}}_{i}+\beta_{2}^{\left[K\right]}\times {\mathrm{SEX}}_{i}$$

All β parameters were estimated from normally distributed prior distributions with a mean of 0 and precision of 0.01. We evaluated the difference in *K* between successional and mature habitats by subtracting the two model coefficients, such that more positive values indicated larger growth coefficients in mature forest subpopulations. We treated the difference as significant if greater than 97.5% of the posterior density was on one side of zero. We ran the growth model on five Markov Chain Monte Carlo (MCMC) chains for 200,000 iterations with a burn‐in of 25,000 and a thinning rate of 5. We considered models to have fully converged if all parameters had Gelman–Ruben (Rhat) statistics below 1.05 and visual inspection of MCMC chains indicated clear and consistent mixing.

#### 2.2.3 Space‐use

Using parameters estimated from our fitted SCR model, we assessed space‐use and overlap in salamanders occupying the successional and mature habitats. Specifically, we plotted each individual's spatial location in coordinate space and then calculated the probability (*p*) of each individual (*i*) using adjacent spatial locations (*j*) as a function of distance (*d*) following a negative exponential function:

$$p_{\mathrm{ijk}}=\exp\left(-{\left(\frac{d_{\mathrm{ijk}}}{\sigma_{k}}\right)}^{2}\right).$$

The rate of probability decay in space is governed by *σ*, which was estimated during the fitting of the SCR model. Probability‐of‐use surfaces were created for each individual at 1000 samples (*k*) of the fitted posterior model distributions. Using the probability surfaces *p*_*ijk*_, we distributed 1000 hypothetical “use” points on the landscape following a random multinomial process. We then calculated kernel density utilization distributions (UD) of these spatially referenced use points for each individual and posterior sample using the R package “adehabitatHR” (Calenge, 2006). Finally, we calculated the probability that the core 50% of each individual *i'*s UD overlapped with all other core 50% UD_*j*_ calculated as the probability of home range overlap (Fieberg & Kochanny, 2005). We then determined the average number of individuals with overlapping core UDs, as well as the average probability of overlap.

#### 2.2.4 Population projection

Using parameters from our fitted individual growth model and from the literature, we conducted population projection simulations to understand how differences in individual growth could impact lifetime fecundity. We assume that all individuals are 13.5 mm SVL upon hatching and that sexual maturity is first reached at 34 mm SVL (Anthony & Pfingsten, 2013). However, following Lotter (1978), we assume that individuals between 34 and 43 mm SVL have 56% chance of being gravid, while 94% of females >43 mm SVL are likely to be gravid (Jaworski et al., 2018; Wise & Jaeger, 2021). Regional variation in reproduction has been documented (Lotter, 1978; Sayler, 1966; Werner, 1971), but our data are not sufficient to ascertain frequency of reproduction in our Ohio population and we therefore use the averages reported by Lotter (1978). Similarly, we could not confidently determine the average number of eggs produced by each female, nor whether there was a size by fecundity interaction. As such, we fit a linear model with a normal distribution to the data reported in Lotter (1978) relating clutch size to SVL using the R package “brms” (Bürkner, 2016). We found that the normally distributed model better fit the data than a generalized model with a Poisson or negative binomial distribution. Like previous demographic projection models of *P. cinereus* (Hernández‐Pacheco et al., 2021; Homyack & Haas, 2009), we assumed eggs have a 90% hatching rate.

We estimated individual growth and lifetime fecundity at each mature forest and successional forest plot location using 100,000 samples from the posterior distributions of our fitted growth model and the clutch size model. Because the survival estimates from our spatial capture–recapture data are unrealistically high (Table 3), we used the average of male and female annual survival estimates and uncertainty from Muñoz, Miller Hesed, et al. (2016). For each individual, at each time step (1 year), we estimated survival as a random binomial process, with the annual probability of surviving being normally distributed with a mean of 0.836 and standard deviation of 0.07 (truncated to 0.4–1.0). The lifespan of wild *P. cinereus* is unknown, but they are generally believed to be long‐lived (Staub, 2016); we projected our model out 20 years.

## 3 RESULTS

Tree species diversity was greater at successional sites (*n* = 9 species) relative to mature sites (*n* = 5), and both sites were dominated by maple species. There was no difference in average DBH between successional and mature sites. There were fewer trees at successional sites (*n* = 57) than at mature sites (*n* = 76), but the greater number of trees present at mature sites was largely driven by maple saplings (Table 1). Successional and mature forest sites had similar soil moisture, air temperature, soil temperature, and leaf litter depth across surveys (Table 2). Surface soil temperatures at successional sites were on average ~1°C warmer than the mature sites, but with much greater variability; mature sites tended to have deeper and more variable leaf litter. Across all plots and surveys, we captured 682 unique salamanders. Of these, we captured 390 salamanders in successional plots (recaptured 114) and 292 salamanders in mature forest plots (recaptured 68). We identified 311 females, 217 males, and 154 juveniles across all plots (Table 2). Overall, observations of salamanders co‐occurring under the same cover board were more than twice as common on mature plots (297) compared with successional plots (135). This equates to 76% of all salamander observations under mature plot cover boards being with another salamander, while only 46% of salamanders were with another salamander under successional plot cover boards. The most frequent co‐occurrence observation among mature salamanders at both mature and successional forest plots was female–female; male–male and male–female co‐occurrences were 2–3 times less common (Table 2).

**TABLE 2:** Habitat characteristics and Eastern Red‐backed Salamander, *Plethodon cinereus*, capture summaries of unique salamanders between the mature and successional forest sites across all sampling seasons (Spring 2017–Fall 2019).

Habitat characteristic	Site	
	Mature	Successional
Soil moisture (% water)	0.266 (0.087)	0.265 (0.094)
Air temperature (°C)	12.050 (6.259)	12.050 (6.220)
Surface soil temperature (°C)	10.198 (4.266)	11.169 (8.660)
Leaf litter depth (cm)	1.979 (4.162)	1.350 (0.818)
Capture summary		
Total captures	390	292
Male/female/juvenile	131/179/80	86/132/74
Recapture percentage	29.2	23.3
Average SVL (mm)	37.73	36.16
Co‐occurrence summary		
All	297	135
Male–male	21	11
Male–female	32	10
Female–female	63	23

*Note*: Habitat values are means (±SD) of measurements collected during each survey (*n* = 17 measurements). Co‐occurrence summaries report the number of times, across all surveys and recaptures, that two or more salamanders were found under the same cover board. Co‐occurrence observations could be male–male, female–female, or male–female; observations with at least one juvenile salamander are included in “All.”

### 3.1 SCR model

For most parameters estimated in our spatial capture–recapture model, mature and successional forest plots had moderate differences (Table 3). Annual survival was estimated to be >0.99 for both successional and mature forest plots. Activity centers of salamander in successional plots shifted slightly more between primary sample periods when compared to mature forest plots (1.376 m vs. 1.241 m, respectively), and successional plot salamanders exhibited greater space‐use (successional = 3.823 m, mature = 3.496 m). The density of salamanders was significantly higher in mature forest plots, which also had significantly higher probability of detection (Table 3).

**TABLE 3:** Parameter estimates from fitted spatial capture–recapture model for *Plethodon cinereus* in central Ohio.

Parameter	Estimate		Probability successional > mature
	Mature Forest	Successional Forest	
Annual survival, Φ	0.996 ± 0.002 [0.99, 0.999]	0.993 ± 0.004 [0.984, 0.999]	0.359
Mean dispersal (m)	1.241 ± 0.146 [0.961, 1.534]	1.376 ± 0.202 [0.995, 1.784]	0.708
Space‐use, σ (m)	3.496 ± 0.164 [3.189, 3.839]	3.823 ± 0.221 [3.419, 4.286]	0.885
Density (per m^2^)	0.613 ± 0.089 [0.558, 0.800]	0.432 ± 0.072 [0.380, 0.580]	0.050
Detection probability, λ	0.019 ± 0.002 [0.016, 0.023]	0.015 ± 0.002 [0.012, 0.019]	0.066

*Note*: Reported values are the mean ± SD with 95% Bayesian credible intervals in brackets. Dispersal represents the average shift in activity centers between seasons, while space‐use represents movement around activity centers. The probability of the successional plot parameter estimate being greater than the mature plot parameter estimate was determined by comparing posterior samples from the fitted model.

### 3.2 Individual growth estimates and time to maturity

**FIGURE 2:**
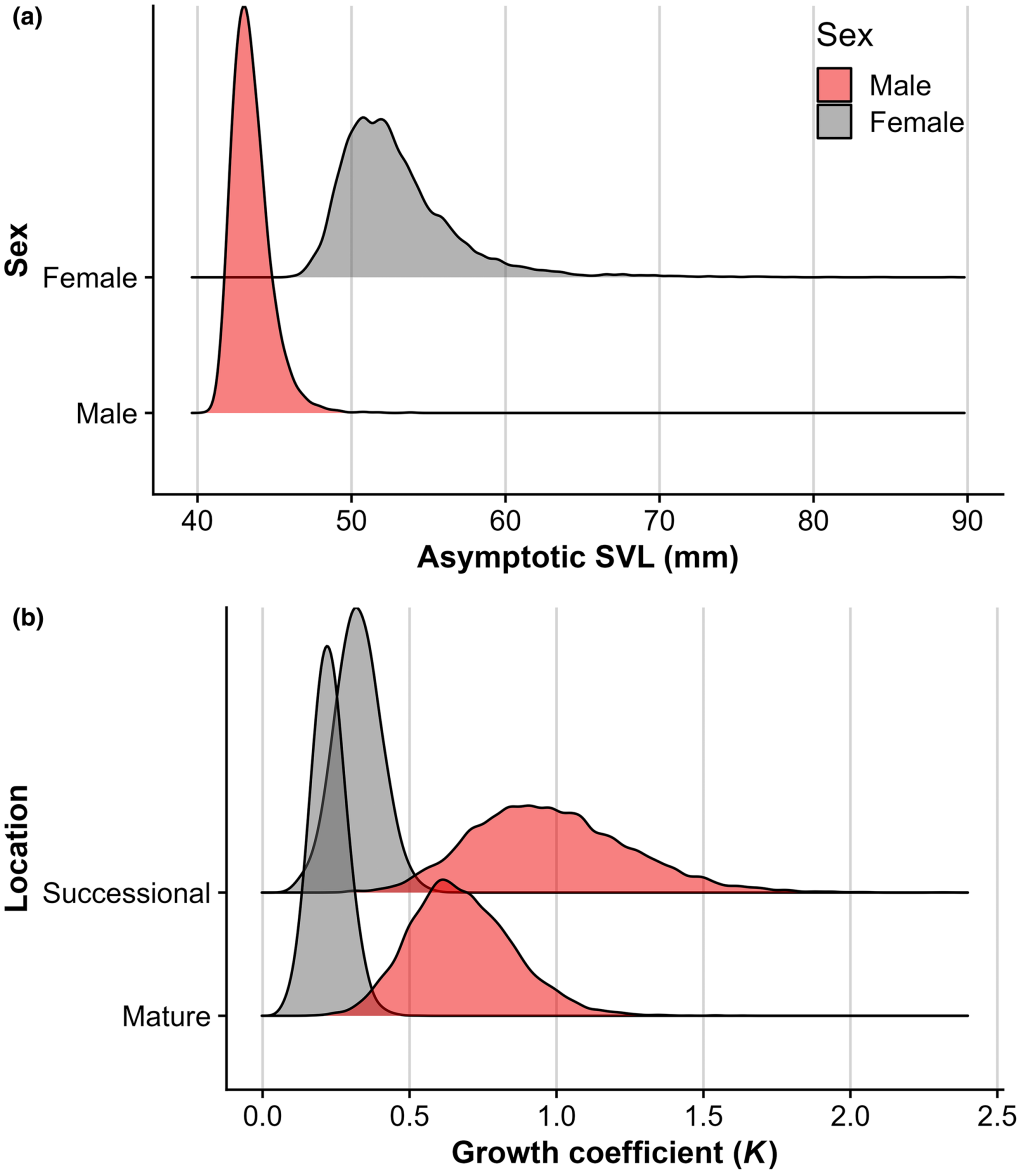
Density plot showing the posterior distributions for asymptotic size for male and female Eastern Red‐backed Salamanders, *Plethodon cinereus* (a) and posterior distributions for the growth coefficient for males and females occupying mature and successional forest plots (b). With greater than 99% probability, all contrasts indicate that males are smaller than females, males grow faster than females, and males and females on the successional forest plots grow faster than males and females on the mature forest plots.

**FIGURE 3:**
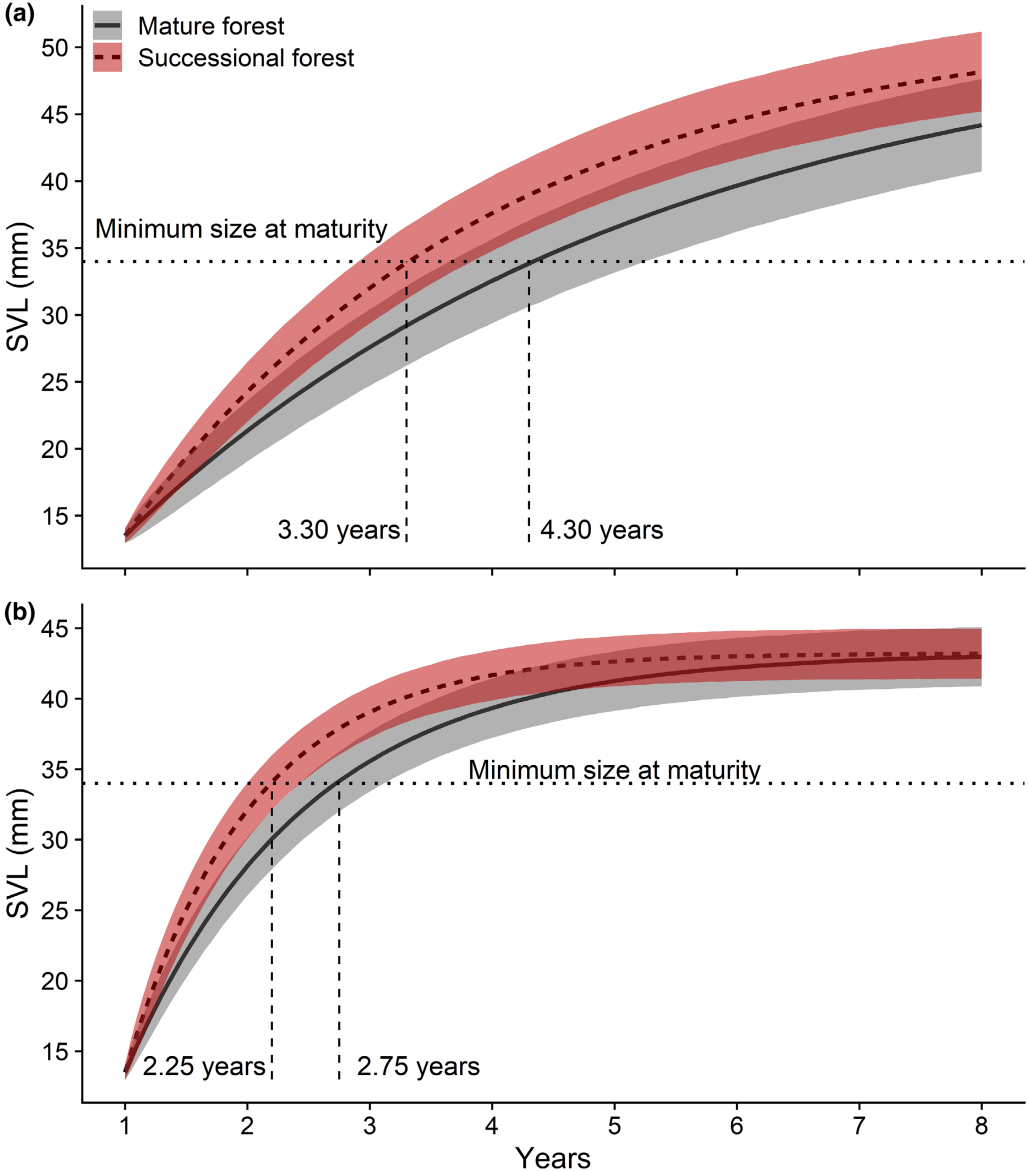
Time to maturity plot, indicating the expected time it would take female (a) and male (b) Eastern Red‐backed Salamanders, *Plethodon cinereus*, to reach sexual maturity (34 mm SVL), given their development in either mature or successional habitats. Starting from a hatching SVL of 13 mm, 50% of juvenile female salamanders occupying successional habitat are expected to reach the minimum size of sexual maturity after 3.30 years compared to 4.30 years for females occupying the mature forest plots (a). By contrast, 50% of males are expected to reach maturity in 2.25 years and 2.75 years in successional and mature forest habitat, respectively (b).

On average, initial salamander mean SVL ± standard deviation was 37.11 ± 4.80 mm, with little difference observed between males (38.00 ± 3.61 mm) and females (38.50 ± 4.47 mm). Similarly, there was no observed difference in the overall mean SVL between mature forest (37.61 ± 4.45 mm) and successional forest (36.43 ± 5.16 mm) plots. However, there were significant differences in asymptotic growth and individual growth rates between males and females and significant differences in individual growth rates between the mature and successional forest locations (Table 4; Figure 2). This results in males in the successional forest reaching sexual maturity in 2.25 years and males in the mature forest maturing in 2.75 years, while females in the successional forest sexually mature in 3.30 years and those in the mature forest sexually reach maturity in 4.30 years (Figure 3).

**TABLE 4:** Parameter description and estimates from the fitted von Bertalanffy individual growth model for *Plethodon cinereus* in central Ohio.

Parameter	Description	Estimate
*L* (male)	Asymptotic size (SVL) of males	43.569 ± 1.319 [41.721, 46.782]
*L* (female)	Asymptotic size (SVL) of females	52.164 ± 2.856 [47.958, 59.062]
*K* (mature, male)	Growth coefficient for males on the slope	0.671 ± 0.176 [0.36, 1.049]
*K* (mature, female)	Growth coefficient for females on the slope	0.237 ± 0.056 [0.137, 0.357]
*K* (successional, male)	Growth coefficient for males on the ridge	0.97 ± 0.266 [0.506, 1.547]
*K* (successional, female)	Growth coefficient for females on the ridge	0.339 ± 0.073 [0.205, 0.489]

*Note*: Reported values are mean ± SD with 95% Bayesian credible intervals in brackets.

### 3.3 Space‐use

**FIGURE 4:**
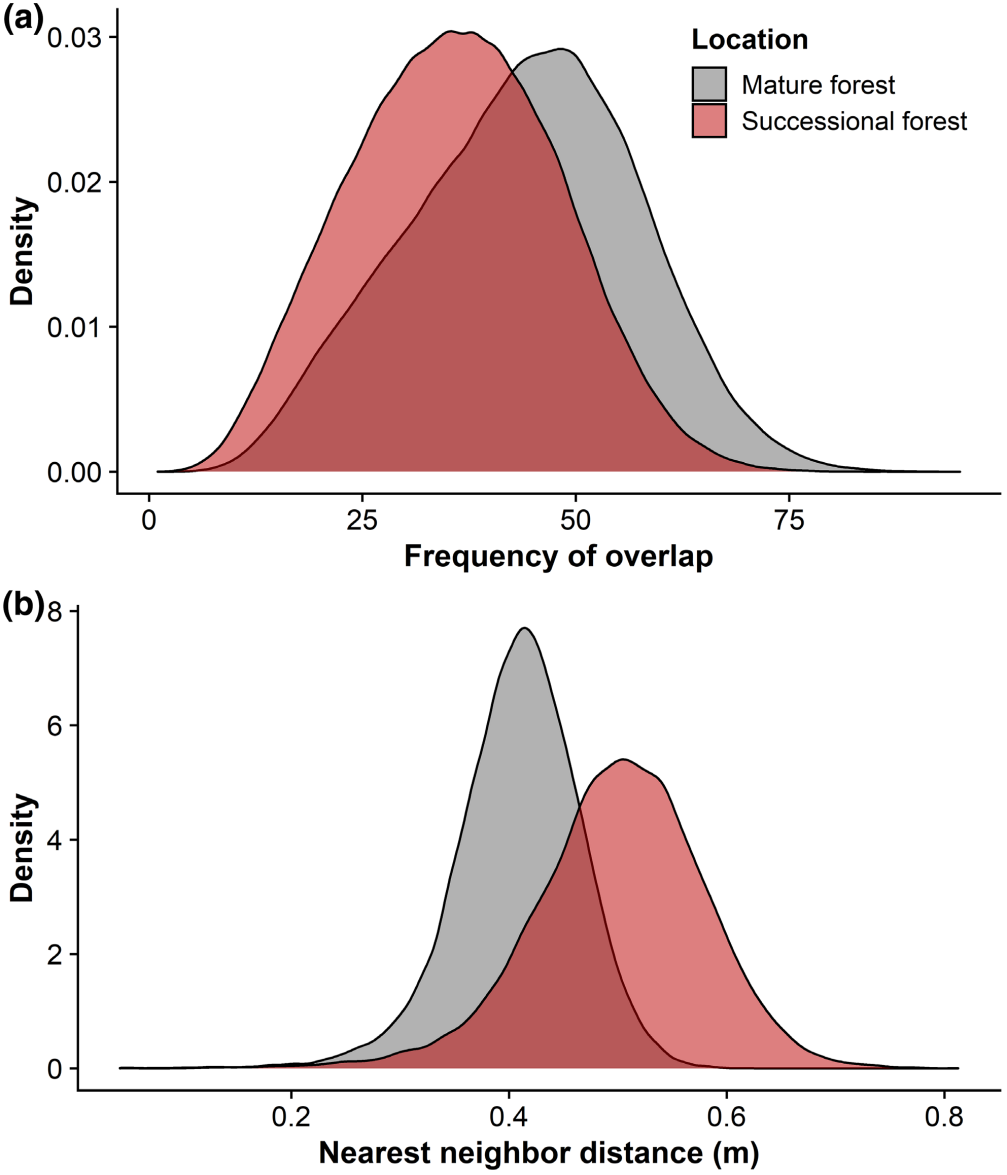
Density plot showing the average number of individual *Plethodon cinereus* that had overlapping core (50% UD) home ranges with each salamander (a) and the average distance between salamander activity centers (b) in mature and successional forest plots. Salamanders occupying mature habitat tended to have more individuals potentially occurring within their core home range than salamanders occupying successional forest habitat, which coincided with less distance between salamanders occupying mature forest plots.

Salamanders occupying successional habitat had substantially fewer individuals overlapping their core UD (35.8 ± 12.1) than salamanders occupying mature habitat (43.8 ± 13.3; Table 5, Figure 4). Despite the greater number of individuals potentially occupying the same space in the mature forest habitat, the average probability of overlap was nearly identical between the two habitats (Table 5). However, the average distance to the nearest neighboring salamander tended to be less for salamanders occupying mature habitat (0.40 m ± 0.06) compared with salamanders occupying successional habitat (0.50 m ± 0.08; Figure 4).

**TABLE 5:** Summary of the space use statistics for *Plethodon cinereus* occupying successional and mature habitats.

Parameter	Estimate	
	Mature	Successional
Mean PHR_*ij*_	0.103 (0.014)	0.099 (0.017)
Max PHR_*ij*_	0.431 (0.042)	0.422 (0.047)
Overlap	43.8 (13.3)	35.8 (12.1)
NN (meters)	0.40 (0.06)	0.50 (0.08)
Average clutches	2.2 (3.7)	2.8 (4.1)
Average fecundity	13.7 (27.3)	19.6 (33.5)

*Note*: Probability of home range overlap (PHR) is reported as both the average for each individual (*i*) relative to all other individuals (*j*) within the same survey plot, as well as the maximum probability of core utilization distribution (UD) overlap. Overlap reports the average number of individuals with overlapping core UD and NN summarizes the average distance to the next closest salamander in the plot. All statistics are means (±SD).

### 3.4 Population projection

Given the annual survival rate estimated from Muñoz, Miller, et al. (2016), females are estimated to live an average of 5.87 years (± 4.90). Females in the mature habitat are estimated to average 2.2 (±3.7) clutches in their lifetime, equating to a projected mean lifetime fecundity of 13.7 (±27.3). Because maturity is reached earlier in the successional habitat, females are estimated to average 2.8 (±4.1) clutches and produce a mean of 19.6 (±33.5) offspring in their lifetime, which is 43% more than females occupying mature forest (Table 5).

## 4 DISCUSSION

Demographic processes, population vital rates, and space‐use are driven by the abiotic and biotic environment experienced by an organism. While variation in these rates is often expected across broad spatial scales (e.g., latitude), our study shows that variation can exist at fine spatial scales between animals occupying different microhabitats and separated by as little as 100 m. In this study, we predicted that there would be differences in salamanders occupying the mature and successional forest plots due to environmental differences between sites. As expected, salamanders in successional forest habitat were present in lower densities and had greater space‐use and activity center shifts than salamanders from mature forest habitat. Salamanders in successional habitat were estimated to be farther from their nearest neighbor and to have less core use overlap with conspecifics than salamanders in mature forest plots. Contrary to our predictions, however, these differences corresponded with salamanders in successional forest growing more rapidly, reaching sexual maturity sooner, and, based on our population projection, having greater projected lifetime fecundity. Ultimately, the processes shaping these patterns remain uncertain as we observed no meaningful differences in soil moisture or air and soil temperature. There were pronounced differences in tree composition and size between sites, but limited differences in leaf litter depth.

We found a higher density of animals with subsequently shorter distances between individuals and greater home range overlap on mature forest plots. The higher proportion of adults on the mature plots, the higher recapture rates on mature plots, and the observed space‐use patterns on mature plots relative to the successional plots are suggestive of territorial adults defending their home range. It is also evident that salamanders encounter each other more frequently at the mature forest plots, but they may not engage in agonistic behaviors due to the energetic costs related to frequent aggressive interactions and allow such overlap to occur. *Plethodon cinereus* and other terrestrial plethodontids are known to reduce agonistic interactions with familiar conspecifics (“dear enemy hypothesis”), especially in areas with high density (Dalton et al., 2020; Jaeger, 1981; Jaeger & Peterson, 2002). The majority of sexually mature salamander co‐occurrence observations in our study were between the same sex and were more prevalent on the higher density mature plots (Table 2). Our home range analyses, in addition to co‐occurrence summaries, suggest differential behaviors of space and cover use at fine scales. In contrast to Hernández‐Pacheco et al. (2019) who found that home ranges are not limited by density, we observed that space‐use was reduced at the higher density mature forest plots. The forest where our mature plots are situated has remained largely undisturbed relative to other central Ohio sites and has the highest observed density of salamanders per square meter when compared to nine other sites in central Ohio, USA (Wilk et al., 2020). Historically, agricultural land uses were more widespread throughout Ohio and eastern North America, with forest cover often increasing with agricultural abandonment (Drummond & Loveland, 2010; Monsted & Matlack, 2021). Our successional plots were historically used as sheep pasture and likely were not widely occupied by *P. cinereus*. As such, mature vs. successional plot differences in our study may reflect habitat quality as well as historical population stability and may be a microcosm of broader trends of forest reversion across Eastern North America and of *P. cinereus*.

The most prominent effect observed in our study was the significant difference in individual growth rates between our plots (Figure 3). Salamanders occupying successional plots grew faster and reached maturity >1 year earlier than individuals occupying mature forest plots. There are at least two possible mechanisms for the observed differences. The first may be related to tree community differences between successional and mature sites (Table 1). Forests across Eastern North America have undergone mesophication and shifted from oak‐dominated tree communities to maple‐dominated (McEwan et al., 2011; Nowacki & Abrams, 2008). Maple leaf litter is often considered higher quality because of the greater proportion of nitrogen to carbon and greater microbial diversity, which is partially responsible for the faster decomposition rates relative to oak (Laking et al., 2021; Lehmann et al., 2020). Such microbial abundance may support a more diverse quality and quantity of prey items available to salamanders (Rittenhouse et al., 2008; Templer et al., 2003) resulting in the faster individual growth and lifetime fecundity observed on the successional plots. Anurans have also been observed to have higher growth and survival when raised in maple‐dominated mesocosms compared with oak (Breslau, 2018). Maple‐dominated forests also tend to be wetter and cooler (McEwan et al., 2011), conditions amenable to lungless *Plethodon* salamanders. While we did not measure notable differences in moisture between our plots, our successional sites had larger maple trees and only one oak, a stark contrast to the 16 large oaks found at the mature sites (Table 1).

A second possible driver, especially in individual growth rate, could be density dependence. Harper and Semlitsch (2007) found that density had a negative effect on survival and growth in metamorphosed American toads (*Anaxyrus americanus*) and wood frogs (*Rana sylvatica*), and Berven (2009) reaffirmed these effects in a long‐term data set of wood frogs. Numerous other studies have identified density dependence in demographic parameters in larval or aquatic urodeles (e.g., Bendik & Dries, 2018; Ousterhout & Semlitsch, 2016; Semlitsch, 1987; Van Buskirk & Smith, 1991), but there is limited research into how density directly affects population demographic parameters of terrestrial plethodontid salamanders. It is important to note, however, that we do not have any estimates of food availability or quality, which should be a focus of future work to better understand the role of density‐dependent processes (Hantak et al., 2016; Kuzmin, 1995).

Regardless of mechanism, the differences in growth rates substantially increase projected lifetime fecundity for females occupying the successional forest plots (Table 5). It is possible there is a greater rate of emigration off successional plots, which may be reflected in our data as we had ~6% lower recapture rate on successional plots as compared to mature forest plots. Spatial capture–recapture models fit to data collected under a robust design allow for the estimation of true rather than apparent survival (Ergon & Gardner, 2014; Gardner et al., 2010; Muñoz, Miller, et al., 2016), but permanent emigration remains an elusive parameter. Emigration in plethodontid salamanders can be particularly challenging as salamanders can temporarily migrate underground or can disperse over land to a new location. During any given survey, only a small fraction of the population is available to be sampled on the surface (Bailey et al., 2004a, 2004b). More research is needed to better understand emigration, especially in seasonally active animals such as *P. cinereus*.

## 5 CONCLUSION

Demography is the most critical driver affecting population persistence (Hanski & Gilpin, 1991). Low reproductive rates, slow maturation, and longer generation times all increase the susceptibility of a population to stochastic events and the potential for local extinction (McKinney, 1997). However, variation in life history traits can buffer populations when environments change (Anderson et al., 2015). *Plethodon cinereus* has proven to be a resilient and adaptable species with a distribution encompassing much of eastern North America and populations frequently persisting in highly altered or urbanized landscapes (Gibbs, 1998; Petranka, 1998; Wilk et al., 2020). The ability to thrive and not just persist in altered or changing habitats may be critical to the species' broad distribution and persistence. We found *P. cinereus* occupying successional habitat to have greater growth rates, which are predicted to result in earlier maturation and greater lifetime fecundity. While observed differences in salamander growth rates and the subsequent demographic differences may be driven by habitat variation and density‐dependent processes, it is also possible for there to be different selective pressures between habitats, leading to microgeorgraphic adaptation. Our results reinforce the role that fine‐scale variation can play in spatial–temporal population processes. Perhaps most notably, these differences occurred between sites <100 m apart, highlighting the importance of accounting for fine‐scale, within‐site variation when assessing demographic processes. As research into the demographic and population consequences of climate change and habitat loss and alteration continue, future research should take care to acknowledge the role that fine‐scale variation in both biotic and abiotic environments may play, especially for organisms with small home ranges or limited mobility.

## AUTHOR CONTRIBUTIONS

**Meaghan Gade:** Conceptualization (supporting); data curation (equal); formal analysis (equal); investigation (equal); methodology (equal); project administration (equal); validation (equal); writing – original draft (equal); writing – review and editing (equal). **Philip Gould:** Conceptualization (supporting); data curation (equal); formal analysis (equal); investigation (equal); methodology (equal); project administration (equal); validation (equal); writing – original draft (supporting); writing – review and editing (equal). **Andrew J. Wilk:** Conceptualization (supporting); data curation (equal); formal analysis (equal); investigation (equal); methodology (equal); project administration (equal); validation (equal); writing – original draft (supporting); writing – review and editing (equal). **Kate C. Donlon:** Conceptualization (supporting); investigation (supporting); methodology (equal); project administration (equal); writing – review and editing (supporting). **Mackenzie L. Brown:** Data curation (supporting); investigation (supporting); writing – review and editing (supporting). **Marnie L. Behan:** Data curation (supporting); investigation (supporting); writing – review and editing (supporting). **Marissa A. Roseman:** Data curation (supporting); investigation (equal); project administration (equal); writing – original draft (supporting); writing – review and editing (equal). **Annalee M. Tutterow:** Data curation (equal); investigation (supporting); writing – review and editing (equal). **Evan D. Amber:** Data curation (equal); formal analysis (equal); investigation (equal); writing – review and editing (equal). **Ryan B. Wagner:** Data curation (supporting); investigation (equal); writing – review and editing (equal). **Andrew S. Hoffman:** Data curation (supporting); investigation (supporting); writing – original draft (supporting); writing – review and editing (equal). **Jennifer M. Myers:** Data curation (supporting); investigation (supporting); writing – review and editing (supporting). **William E. Peterman:** Conceptualization (lead); data curation (equal); formal analysis (equal); funding acquisition (lead); investigation (lead); methodology (lead); project administration (lead); resources (lead); supervision (lead); validation (equal); visualization (lead); writing – original draft (lead); writing – review and editing (equal).

## CONFLICT OF INTEREST

The authors do not have any competing interest.

## Data Availability

Data and code used in this manuscript have been archived at Figshare: https://doi.org/10.6084/m9.figshare.21841590.v1.
